# Aberrant resting-state regional activity in patients with postpartum depression

**DOI:** 10.3389/fnhum.2022.925543

**Published:** 2023-01-19

**Authors:** Bo Li, Shufen Zhang, Shuyan Li, Kai Liu, Xiaoming Hou

**Affiliations:** ^1^Department of Radiology, The 960th Hospital of the PLA Joint Logistics Support Force, Jinan, China; ^2^Department of Obstetrics, Shandong Second Provincial General Hospital, Jinan, China; ^3^Foreign Languages College, Shandong University of Traditional Chinese Medicine, Jinan, Shandong, China; ^4^Department of Pediatrics, Provincial Hospital Affiliated to Shandong First Medical University, Jinan, Shandong, China

**Keywords:** postpartum depression, regional functional connectivity, ReHo, fMRI, functional connectivity

## Abstract

**Background:**

Postpartum depression (PPD) is a common disorder with corresponding cognitive impairments such as depressed mood, memory deficits, poor concentration, and declining executive functions, but little is known about its underlying neuropathology.

**Method:**

A total of 28 patients with PPD and 29 healthy postpartum women were recruited. Resting-state functional magnetic resonance imaging (rs-fMRI) scans were performed in the fourth week after delivery. Individual local activity of PPD patients was observed by regional homogeneity (ReHo) during resting state, and the ReHo value was computed as Kendall's coecient of concordance (KCC) and analyzed for differences between voxel groups. Correlations between ReHo values and clinical variables were also analyzed.

**Result:**

Compared with healthy postpartum women, patients with PPD exhibited significantly higher ReHo values in the left precuneus and right hippocampus. ReHo value was significantly lower in the left dorsolateral prefrontal cortex (dlPFC) and right insula. Furthermore, ReHo values within the dlPFC were negatively correlated with the Edinburgh PPD scale (EPDS) score. The functional connectivity (FC) of the right hippocampus to the left precuneus and left superior frontal gyrus (SFG) was stronger in patients with PPD than that in controls.

**Conclusion:**

The present study provided evidence of aberrant regional functional activity and connectivity within brain regions in PPD, and it may contribute to further understanding of the neuropathology underlying PPD.

## 1. Introduction

Postpartum depression (PPD) has been reported in 10–20% of new mothers within the first 4 weeks following delivery, which is a major public health concern that has significant consequences for mothers, their children, and their families (Gress-Smith et al., 2012). Rates of hospitalization, self-harm, and maternal suicide are increased among depressed women during the peripartum period (Lindahl et al., 2005). Infants with mothers affected by PPD are more likely to suffer infant abuse or infanticide, less infant weight gain, and increased rates of hospitalization (Gress-Smith et al., 2012). PPD further harms infants in their subsequent development of cognition, emotion, and behavior during childhood and adolescence (Gress-Smith et al., 2012).

Although the devastating sequelae of PPD have been comprehensively studied, the diagnosis and pathophysiology of this disease, especially its neuropathology, are not well defined (Duan et al., 2017). Findings from clinical studies and laboratory rodent models highlight that alterations in activation of brain areas during PPD likely alter key neural networks associated with women's maternal care, empathy, stress, motivation, emotional reaction to stimulus valence, learned reward, and executive functioning (Pawluski et al., 2017). As the most important and useful tool to noninvasively study the functions of the brain, functional magnetic resonance imaging (fMRI) has become more commonly used. Physicians use fMRI to detect cerebral blood-oxygenation-level dependent (BOLD) activity in response to changes in neural activity (Logothetis et al., 2001) either with activation or at rest. The studies using fMRI with activation were to investigate differences in mothers' brain responses to infant and non-infant cues. Neural activation between PPD and healthy mothers differs in response to infant- and non-infant-related cues, such that activity in the specific brain regions will increase in response to a non-infant emotional cue but decrease in response to an infant-related emotional cue (Moses-Kolko et al., 2010; Silverman et al., 2011; Barrett et al., 2012; Laurent and Ablow, 2012b; Wonch et al., 2016).

The other studies used resting-state fMRI (rs-fMRI) to analyze women's brain resting state. An advantage of rs-fMRI is that it can clarify how PPD may affect a mother's baseline brain activity at rest and provide a comprehensive understanding of neural circuitry dysfunction in mothers with PPD. Rs-fMRI has been applied to detect spontaneous neural brain activity and functional connectivity (FC) in PPD using the resting-state functional connectivity (RSFC) (Deligiannidis et al., 2013, 2019; Chase et al., 2014), the dynamic amplitude of low-frequency fluctuations ALFF analysis (Cheng et al., 2022b), regional homogeneity (ReHo) analysis (Xiao-Juan et al., 2011), voxel-mirrored homotopic connectivity (Zhang et al., 2020), dynamic functional connectivity (FC) (Cheng et al., 2022b), functional connectivity density (FCD) (Cheng et al., 2021), and functional connectivity strength (FCS) (Cheng et al., 2022a). At rest, women with PPD showed decreased corticocortical and corticolimbic connectivity. More specifically, the women with PPD showed significantly weaker connectivity among the amygdala (AMG), anterior cingulate cortex (ACC), dorsal lateral prefrontal cortex (dlPFC), and the hippocampus compared with non-depressed postpartum women (Deligiannidis et al., 2013). In addition, they showed negative connectivity between the posterior cingulate cortex (PCC) and AMG (Chase et al., 2014). The area of the dorsomedial prefrontal cortex (dmPFC) has greater connectivity with the rest of the default mode network (DMN) and reduced connectivity with the precuneus, posterior cingulate cortex, and supramarginal gyrus/angular gyrus regions in women with PPD (Deligiannidis et al., 2019). PPD mothers exhibited increased FC between the subgenual anterior cingulate cortex (sgACC) and ventral anterior insula and disrupted FC between the sgACC and middle temporal gyrus. The changes in dynamic FC between the sgACC and superior temporal gyrus could differentiate PPD and HCs (Cheng et al., 2022b). Patients with PPD showed specifically weaker long-range FCD in the right lingual gyrus (L.G.R), functional couplings between LG.R and dmPFC, and left precentral gyrus and specifically stronger functional coupling between LG.R and right angular. Moreover, the altered FCD and RSFC were closely associated with depression and anxiety symptoms load (Cheng et al., 2021). PPD group showed specifically higher FCS in right parahippocampus, and perceived social support mediated the influence of FCS in the right cerebellum posterior lobe on depression and anxiety symptoms (Cheng et al., 2022a). Compared with healthy controls (HCs), mothers with PPD showed significantly increased posterior cingulate and medial frontal gyrus and decreased temporal gyrus ReHo (Xiao-Juan et al., 2011). Patients with PPD exhibited significantly decreased voxel-mirrored homotopic connectivity values in the bilateral dmPFC, dorsal anterior cingulate cortex (dACC), and orbitofrontal cortex (Zhang et al., 2020). Compared to the relative wealth of data available for major depression, fMRI studies of PPD were limited in number and design. It seems reasonable to conduct studies using a variety of rs-fMRI techniques to help identify neuroimaging signatures for PPD.

In this study, we focused on the homogeneity of regional activity by investigating changes in ReHo, which is distinct from mainstream methods of measuring long-range connections using the amplitude of low-frequency fluctuations. ReHo is a highly sensitive, reproducible, and reliable index of local activity and can reflect functional similarities in brain activities among neighboring voxels located within a short range (Ji et al., 2020). Decreases or increases in ReHo are thought to, respectively, reflect spontaneous neural hypoactivity or hyperactivity in a given regional brain area (Jiang and Zuo, 2016). Here, we collected a dataset from 57 participants, including 28 patients with PDD and 29 HCs. We calculated individual local activity by ReHo and explored their potential correlations with the clinical symptoms. We further used the brain areas with abnormal ReHo values in PPD as the region of interest (ROI) and conducted FC analysis. We tested the following hypotheses: (1) the PPD group showed abnormal ReHo in DMN and limbic system compared to HCs; (2) the alterations of ReHo would be related to Edinburgh postpartum depression scale (EPDS) score; and (3) the brain regions with abnormal ReHo in DMN and the limbic system showed the aberrant FC.

## 2. Material and methods

### 2.1. Participants

This study included 57 participants (28 patients with PDD and 29 HCs) who were recruited from the Department of Obstetrics of Shandong Second Provincial General Hospital and the Department of Obstetrics of the 960th Hospital of the PLA Joint Logistics Support Force. Two experienced senior associate chief physicians of neurology confirmed their diagnoses by using the Structured Clinical Interview for Diagnostic and Statistical Manual of Mental Disorders, Fifth Edition (DSM-5) and Chinese Classification and Diagnostic Criteria of Mental Disorders, 3rd edition (CCMD-3). Inclusion criteria for patients were as follows: new mothers (a) whose age ranged from 21 to 38 years, in the fourth week after delivery; (b) with first-episode, treatment-naive PPD patients; (c) with EPDS score of ≧ 13; (d) with no other medical or mental illness history; (e) with no substance abuse or substance dependent; (f) with no contraindications of an MR examination; and (g) with no organic abnormalities for MRI routine series. The EPDS score was assessed 1 h before the image acquisition. Inclusion criteria for HCs were as follows: new mothers (a) whose age ranges from 21 to 38 years, in the fourth week after delivery; (b) with no current or previous history of depressive episodes; (c) with EPDS score < 3; and (d)–(g) were same to the PPD group. This study was approved by the ethics committee of the Shandong Second Provincial General Hospital and all participants provided written informed consent.

### 2.2. Image acquisition

All fMRI data were acquired on a 3.0T MR system (Discovery MR750, General Electric, Milwaukee, USA) with a standard eight-channel head coil. During scanning, all participants were instructed to lie quietly and remain still with their eyes closed and heads fixed in place by foam pads to minimize head movement.

High-resolution structural T1-weighted scans (Three-dimensional Brain Volume, 3D BRAVO) were performed using the following parameters: time repetition (TR) = 8.2 ms, time echo (TE) = 3.2 ms, flip angle = 12°, the field of view (FOV) = 240 mm × 240 mm, slices = 115, voxel size = 1 mm, and thickness = 1.0 mm. Resting-state BOLD MR images were acquired with the following parameters: T*R* = 2,000 ms, TE= 40 ms, flip angle = 90°, FOV = 240 mm × 240 mm, resolution = 64 × 64, voxel size = 3.75 mm, thickness = 4.0 mm, no interspace, slices = 41, gradient echo-planar volumes = 200, and duration was 6 min 40 s. In addition, routine MRI data were collected to exclude anatomic abnormality among all participants.

### 2.3. Data preprocessing

The fMRI data preprocessing was conducted using the Data Processing Assistant for rs-fMRI (DPARSF) and rs-MRI data analysis toolkit (REST) (http://www.restfmri.net), which are based on Statistical Parametric Mapping (SPM12; http://www.fil.ion.ucl.ac.uk/spm). First, the first ten volumes were discarded. Second, the slice-time corrected images were realigned to the first volume for slice-timing correction. For head motion correction, all subjects with a head motion > 1.5° rotation and 1.5 mm translation were excluded. For the frame-wise displacement estimates, we used a volume censoring technique (“scrubbing”) (Power et al., 2012) to eliminate the potential impact of sudden motions or moderate motions on the FC. We normalized motion-corrected functional images to a standard EPI template in the Montreal Neurological Institute space by applying the parameters of structural image normalization and resampling the normalized images to 3 mm isotropic voxels. After linear detrend, the data were band-pass filtered (0.01–0.08 Hz) to eliminate physiological noise. Several sources of spurious covariates along with their temporal derivatives, including the six head motion parameters, global mean, white matter, and cerebrospinal fluid, were removed.

### 2.4. ReHo analysis

The ReHo value was computed as KCC in rs-fMRI (Zang et al., 2004). A KCC was assigned to a given voxel by calculating the KCC of the time series of this voxel with 26 nearest neighbors. A higher KCC indicates a higher synchronization. We obtained the standardized KCC value by dividing the KCC value of each voxel by the average value of the whole brain. The KCC program was coded in MATLAB (The MathWorks, Inc., Natick, MA). Thus, an individual ReHo map was generated for each subject. Finally, we smoothed all individual ReHo maps by using a 4-mm full-width at half-maximum Gaussian kernel.

The variables, including age and clinical symptom scores between PPD and the control group, were analyzed by the Mann–Whitney *U*-test in SPSS version 18.0 (SPSS Inc., Chicago, IL, USA). The differences in the delivery method were determined using chi-square tests. The threshold was set at *p* < 0.05. With age as a covariate, a two-sample *t*-test was performed using the REST1.8 software to determine significant voxel-based differences in ReHo value between the two groups. The resulting statistical map was set at *q* < 0.05 for multiple comparisons (FDR corrected, cluster size > 55 voxels) using the REST1.8 software.

### 2.5. FC analysis

We used the brain clusters with significantly different ReHo values between PPD and HCs as the ROIs. FC analyses were performed using the default FC processing pipeline in the REST toolbox. In this processing pipeline, white matter, cerebral spinal fluid noise, and global mean signal were removed through regression after spatially smoothing (4-mm full-width at half-maximum). The mean time series from each ROI was calculated by averaging the time series of all the voxels within that region. The seed-based FC analysis was computed between the seed reference time course and that of each voxel in the brain in a voxel-wise way. Finally, the Fisher r-to-Z transformation was used to transform correlation coefficients to Z values. FDR correction (*q* < 0.05) was performed for multiple comparisons.

### 2.6. Correlation analysis between the brain and clinical characteristics

In this study, we performed a correlation analysis between clinical characteristics and brain functional metrics, including ReHo and FC strength. The mean ReHo in each between-group significant cluster was extracted for each subject. After seed-based FC analysis, the mean FC within each between-group significant cluster was extracted. Finally, Pearson correlation analyses were performed between the mean ReHo or FC strength in each cluster and the EPDS scores in the PPD with HCs group using the SPSS version 18.0 software with significance at *p* < 0.05.

## 3. Results

### 3.1. Demographic and clinical characteristics

A total of 28 PPD patients and 29 HCs were enrolled in the final analyses of this study. All the participants were right-handed. We found no significant differences in age, educational level, delivery method, delivery time, or feed options among the PPDs and controls. Patients with PPD showed significantly higher EPDS scores than HCs (*t* = 36.514, *p* < 0.001), as shown in Table 1.

**Table 1:** Demographic factors and clinical data.

**Characteristic**	**Healthy control (HC**, ***n =*** **29)**		**Postpartum depressed (PPD**, ***n =*** **28)**		***p*-value**
	**Mean (SD)**	**Percent (%)**	**Mean (SD)**	**Percent (%)**	
Age (years)	28.56 (4.57)		29.27 (4.72)		0.82
Right handedness	29	100	20	100	
Socioeconomic status	181.64 (4.60)		182.84 (3.26)		0.26
(thousand RMB)					
Education (years)	11.28 (3.74)		11.85 (3.26)		0.60
Cesarean	10	34.5	11	39.2	0.87
Breastfeeding	29	100	28	100	
Primi para	14	48.3	15	53.6	0.90
EPDS	0.79 (0.96)		14.97 (1.66)		0.00

SD, standard deviation; RMB, Renminbi; EPDS, Edinburgh postpartum depression scale.

### 3.2. Intergroup differences in ReHo values

**Figure 1:**
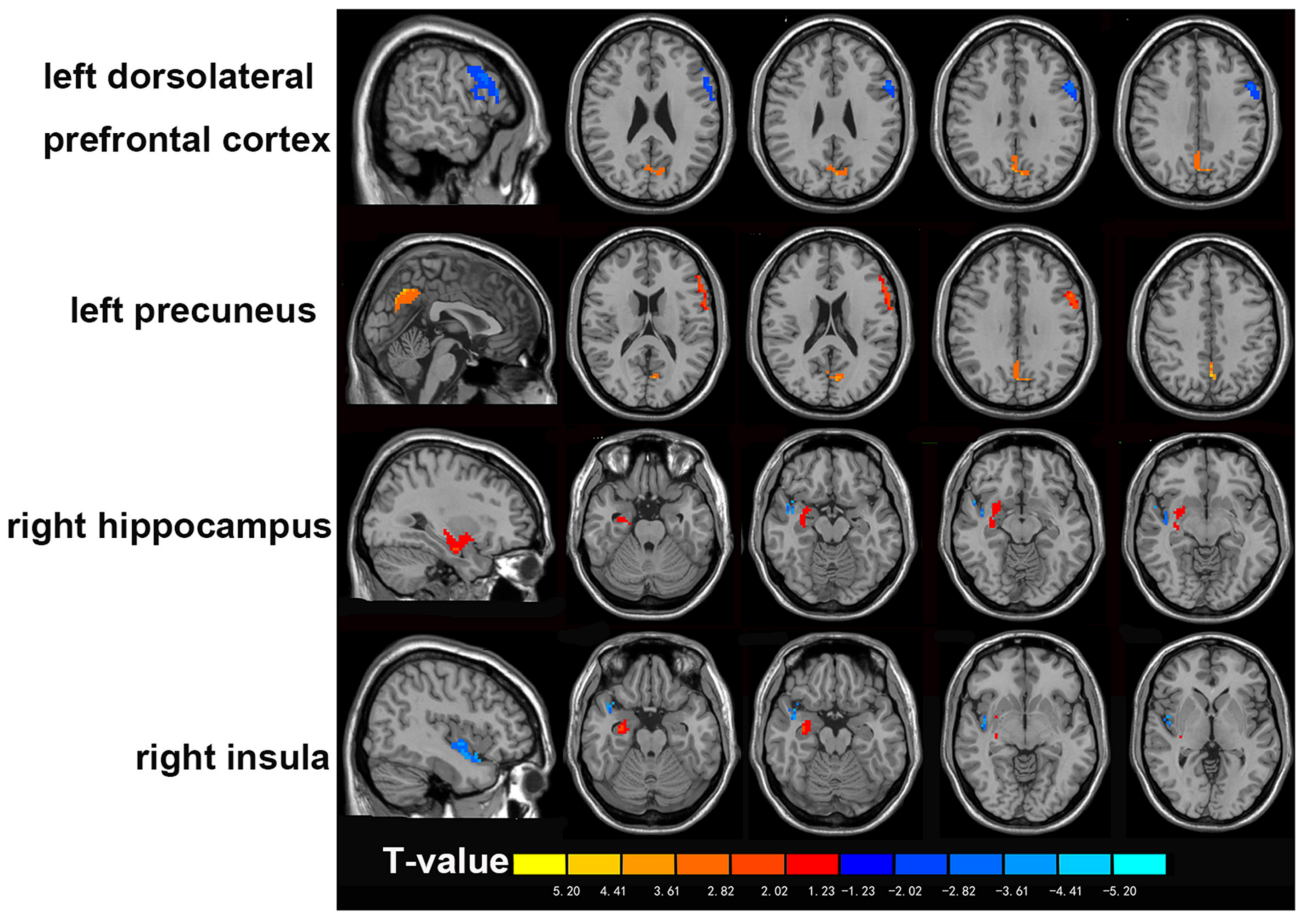
Comparisons of rs ReHo values between patients with PPD and HCs. The PPD group exhibited higher ReHo in left precuneus and right hippocampus. ReHo was significantly lower in left dlPFC and right insula. Warm color represents significantly increased ReHo, and cool color represents significantly decreased ReHo. dlPFC, dorsolateral prefrontal cortex; HCs, healthy controls.

The PPD group exhibited higher ReHo in the left precuneus and right hippocampus. ReHo was significantly lower in the left dlPFC and right insula (Figure 1). Specific ReHo values of the PDD groups are listed in Table 2.

**Table 2:** Regional homogeneity values in brain regions showing significant group differences.

**Brain region**	**Peak MNI coordinates**			**Cluster size (mm^3^)**	**Peak *T*-value**	**ReHo** **direction**
	**x**	**y**	**z**			
Left dorsolateral	−54	12	30	190	−3.07	PPD < HC
prefrontal cortex						
Right insula	42	−3	−15	55	−3.75	PPD < HC
Left precuneus	−1	−63	42	120	4.52	PPD > HC
Right hippocampus	33	−12	−18	89	2.23	PPD > HC

MNI, Montreal Neurological Institute.

### 3.3. Correlations between ReHo and clinical characteristics

**Figure 2:**
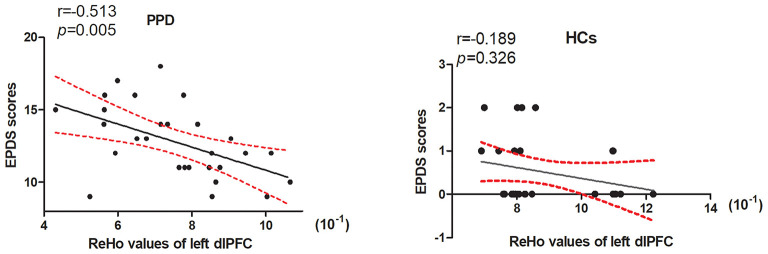
The ReHo values within the left dlPFC were significantly negatively correlated with the EPDS scores in the PPD group (*r* = −0.513, *p* = 0.005). There were no significant correlations between the ReHo values within the left dlPFC and EPDS scores in HCs group (*r* = −0.189, *p* = 0.326). dlPFC, dorsolateral prefrontal cortex; EPDS, Edinburg postpartum depression scale; HCs, healthy controls.

The ReHo values within the left dlPFC were significantly negatively correlated with EPDS scores in the PPD group (*r* = −0.513, *p* = 0.005) (Figure 2). There were no significant correlations among the ReHo values in any other regions and EPDS scores in PPD and HC groups.

### 3.4. Seed-based FC analysis

**Figure 3:**
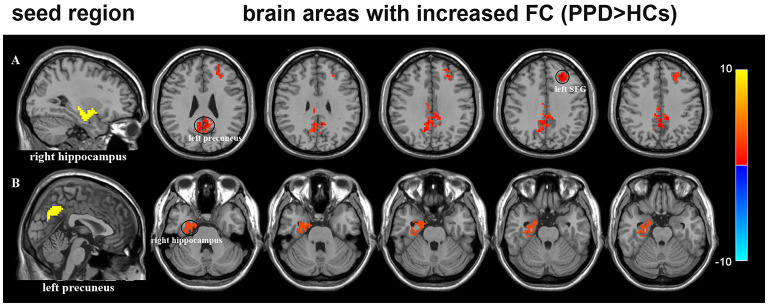
**(A)** Brain regions showing aberrant FC with right hippocampus (seed region) in the PPD group compared with HCs. Warm color represents significantly increased FC. The FC of the right hippocampus to the left precuneus and left SFG was stronger in patients with PPD than in HCs. **(B)** Brain regions showing FC with left precuneus (seed region) in the PPD group compared with HCs. Warm color represents significantly increased FC. The FC of the left precuneus to right hippocampus was stronger in patients with PPD than in controls. FC, functional connectivity; HCs, healthy controls; SFG, superior frontal gyrus.

We used four brain regions (left dlPFC, left precuneus, right hippocampus, and right insula) with significantly different ReHo values between PPD and HCs as seeds in the FC analysis of the whole brain. In the PPD group, the right hippocampus (seed region) showed increased FC with the left precuneus and left superior frontal gyrus (SFG) compared with HCs. The left precuneus (seed region) showed increased FC with the right hippocampus compared with HCs (Figure 3 and Table 3). There were no other significantly different FCs in any ROIs with the whole brain between the two groups.

**Table 3:** Significant differences in FC between PPD and HCs.

**Seed region**	**Area with altered FC**	**Peak MNI coordinates**			**Cluster size (mm^3^)**	**Peak *T*-value**	**FC direction**
		**x**	**y**	**z**			
Right hippocampus	Left precuneus	−6	−51	30	268	4.35	PPD > HC
	Left superior frontal gyrus	−24	33	36	104	4.03	PPD > HC
Left precuneus	Right hippocampus	26	−5	−23	109	4.67	PPD > HC

### 3.5. Correlations between FC and clinical characteristics

There were no significant correlations among the FC values in any regions and EPDS scores in PPD and HC groups.

## 4. Discussion

This study measured the ReHo value using rs-fMRI and correlations among abnormal ReHo values within regions and clinical characteristics in patients with PPD. In this study, we observed the following: (1) ReHo value was higher in the left precuneus and right hippocampus and lower in the left dlPFC and the right insula in the PPD group; (2) ReHo value within the left dlPFC was significantly negatively correlated with EPDS scores in the PPD group, with age as covariates; and (3) the right hippocampus showed increased FC with the left precuneus and left SFG compared with HCs.

The precuneus is a key component of the DMN (Utevsky et al., 2014) and a critical hub with dense and widespread connectivity in human whole-brain structural and functional networks. The precuneus plays a pivotal role in a wide spectrum of functions, including cognition, memory retrieval, self-consciousness, visuospatial imagery, emotional judgment, and self-referential processing (Cavanna and Trimble, 2006). The alterations in the precuneus might be associated with thought-action fusion disturbance, self-referential processing ruminations, and the dysregulation of the sensory part of the fear network, which might be the putative biomarker for depression and anxiety (Lai, 2018). The higher ReHo in the left precuneus and the stronger FC between the precuneus and hippocampus were found in patients with major depressive disorder (MDD) (Yang et al., 2015; Cheng et al., 2018; Xiao et al., 2021). In this study, we found higher ReHo in the left precuneus and stronger connectivity between the left precuneus and right hippocampus in patients with PPD. The earlier fMRI studies of PPD showed a negative coupling between the precuneus and right AMG region and dmPFC (Chase et al., 2014; Deligiannidis et al., 2019). The abnormal function and connectivity in the left precuneus might explain depression and anxiety among PPD.

As the core region in the limbic system and DMN, the hippocampus plays a very important role in memory, regulation of motivation, stress, and emotion (Eichenbaum, 2013). Patients with MDD have shown to have impaired FCs of the hippocampus, which might explain the memory deficits and depression experienced by patients with MDD (Hao et al., 2020). We observed decreased FC in the left hippocampal-ROI to the bilateral middle frontal gyrus, as well as in the right hippocampal-ROI to the right inferior parietal cortex (IPC) and the cerebellum in patients with MDD compared to the HCs (Cao et al., 2012). Here, we found higher ReHo in the right hippocampus in PPD and stronger connectivity between the left precuneus and right hippocampus. Subjects with PPD had already shown the attenuation of connectivity between the dlPFC and hippocampus (Deligiannidis et al., 2013). The abnormal activity and functional connections of the hippocampus might also be evidence of the depression experienced by patients with PDD.

It is worth noting that both the left precuneus and right hippocampus are important regions in DMN (Zhang et al., 2021). Altered spontaneous neural activities and altered FC between the left precuneus and right hippocampus indicate DMN dysfunction in patients with PPD. It is known that depression symptoms are associated with excessive self-focus, a tendency to engage oneself in self-referential processing (Mor and Winquist, 2002). DMN is responsible for spontaneous cognition, self-referential processing, and emotional regulation (Ho et al., 2015). After taking this evidence into consideration, it is hypothesized that aberrant DMN function may lead to self-referential processing abnormally integrating with biased emotional memory in PPD. Failure of DMN deactivation during emotional or cognitive tasks has been proposed as a possible mechanism acting in PPD.

In the frontal lobe, the dlPFC acts as a key node of the brain networks, including the extrinsic mode network (Hugdahl et al., 2015) and cognitive control network (Cole and Schneider, 2007). It has been implicated in cognitive, affective, sensory processing, and emotional regulation, such as attention, value encoding (Liu et al., 2016), working memory, creativity (Liu et al., 2015), decision-making (Rahnev et al., 2016), reappraisal, expectation, and desire for relief (Sevel et al., 2016). Functional imaging studies have shown decreases in regional cerebral blood flow and metabolism in dlPFC, especially on the left side among patients with depression (Dolan et al., 1993). SFGs are a crucial part of the dlPFC (Xiong et al., 2019). The SFG is generally considered a core brain region in the cognitive control system (Niendam et al., 2012) for emotion regulation-related processes (Frank et al., 2014), which are influential factors for depressive symptoms. In this study, we reported that patients with PPD had lower ReHo in left dlPFC than HCs and stronger connectivity between left SFG and right hippocampus. Previous studies have shown that women with PPD showed significantly weaker connectivity among the AMG, ACC, dlPFC, and hippocampus than HCs (Deligiannidis et al., 2013). We also found significant correlations between ReHo values in dlPFC regions and EPDS scores in the PPD group. Higher EPDS scores correlated with lower ReHo values in dlPFC regions. Based on the previous studies (Mayberg, 1997; Mayberg et al., 1999), depression is associated with increased activation in the subcortical and ventral frontal system (subgenual cingulate, ventral insula, hippocampus, ventral frontal, and hypothalamus implicating in the production of normal and abnormal affective states) and relatively decreased activation in the dorsal frontoparietal system (e.g., dlPFC, dorsal ACC, IPL, and PCC implicating in the cognition and regulating the parameters of affective states) (Xiong et al., 2019). This process may be derived from the bottom-up pathway from the limbic areas, through the cingulate and subcortical regions, to the PFC and frontal lobe finally (Disner et al., 2011). Although we have observed stronger connectivity between left SFG and right hippocampus in PPD, inhibited activations of high-order regions (specifically in dlPFC) attenuate the cognitive control of the top-down system and allow the bottom-up hyperactivation (hippocampus) preserved. In addition, the hypoactivation of the dlPFC has been related to an impairment of cognitive control that may favor rumination, which is considered one of the key factors in the onset and maintenance of depression. All results suggested that many symptom profiles of patients with PPD, such as depression and impaired concentration, might be due to the hypofunction of the left dlPFC.

The human insula is implicated as a major multimodal network hub with connections to the frontal, parietal, temporal, and limbic areas (Dionisio et al., 2019). It is a key component of the fronto-limbic circuit and involves a large variety of complex functions, including pain, cognition, memory, emotion, and self-recognition (Augustine, 1996; Nieuwenhuys, 2012). The right insula is involved in sensory function, pain, and saliency processing (Dionisio et al., 2019). The altered functional activity and connectivity of the insula have been observed in patients with depression (Iwabuchi et al., 2014). Patients with MDD have been shown to have reduced ReHo in the right insula (Liu et al., 2010), which correlated with anxiety and hopelessness (Yao et al., 2009). We found lower ReHo in the right insular of patients with PPD. This is the first study that has found the reduced activity of the right insula in PPD by rs-fMRI. In task-based fMRI studies, the mothers with PPD showed decreased insula activation in response to cries (vs. non-cry control and other infant cries), emotional faces (vs. cries/emotional faces of other infants) (Laurent and Ablow, 2012a), and expressions of joy (Fiorelli et al., 2015). These patients showed increased insular activity, especially on the right side, when exposed to negative words or negative stimuli (Silverman et al., 2007). It was also observed that mothers with PPD had decreased AMG-right insular cortex connectivity when viewing their infants compared to other infants (Duan et al., 2017). Therefore, the PPD group may have impaired function in this region. Due to the insula's wide range of functions regarding pain, emotional processing, memory, attention, and cognition, the decline of right insular activity might be related to the PPD's diverse symptoms.

Many brain regions such as the hypothalamus, AMG, anterior cingulate, orbitofrontal cortex, and dlPFC, as well as the insula and striatum, have been shown to be involved in the pathogenesis of PPD and are also linked to mothering (Stickel et al., 2019). Our study showed abnormal activities in the left dlPFC, left precuneus, right hippocampus, and right insula in patients with PPD, which were in accordance with the reported regions. We also noticed an earlier study that showed different brain regions with abnormal ReHo including the posterior cingulate, medial frontal, and temporal gyrus (Xiao-Juan et al., 2011). Our study was conducted with sample sizes over two times larger than the earlier study (10 patients with PPD). Furthermore, we conducted the seed-based FC analysis and found the right hippocampus showed increased FC with the left precuneus and left SFG in the PPD group compared with HCs. All our results point to a plausible underlying functional foundation of the neural mechanism in the course of PPD. The four regions with abnormal ReHo values and the altered FC were associated with many brain networks including DMN, fronto-limbic circuit system, and cognitive control network. Our results might suggest the abnormal neuro-activity in these brain networks in patients with PPD, which might help better understand the underlying neuropathology of the disease. However, the present study had some limitations. First, we need additional recruitment and further exploration to verify our results. Second, the study lacked the comparison between the pre- and post-treatment of PPD patients and could not provide the imaging change of the above brain areas after treatment. Third, the study lacked participants' other demographic factors that might influence the results of the study, such as smoking, number of pregnancies, and the presence of underlying diseases. In conclusion, our study provided evidence of aberrant ReHo and FC within brain regions in PPD, and it may contribute to identifying the neuroimaging signatures of patients with PPD for diagnosis and a better understanding of the neuropathology underlying PPD.

## Data availability statement

The raw data supporting the conclusions of this article will be made available by the authors, without undue reservation.

## Ethics statement

The studies involving human participants were reviewed and approved by the Ethics Committee of the Shandong Second Provincial General Hospital. The patients/participants provided their written informed consent to participate in this study.

## Author contributions

BL and XH: conception and study design. SZ, BL, and KL: data collection or acquisition. SZ and BL: statistical analysis. XH: interpretation of results and drafting the manuscript work or revising it critically for important intellectual content. SL: revising the manuscript. All authors contributed to the article and approved the submitted version.
